# Large Scale Expression Changes of Genes Related to Neuronal Signaling and Developmental Processes Found in Lateral Septum of Postpartum Outbred Mice

**DOI:** 10.1371/journal.pone.0063824

**Published:** 2013-05-22

**Authors:** Brian E. Eisinger, Changjiu Zhao, Terri M. Driessen, Michael C. Saul, Stephen C. Gammie

**Affiliations:** 1 Department of Zoology, University of Wisconsin-Madison, Madison, Wisconsin, United States of America; 2 Neuroscience Training Program, University of Wisconsin-Madison, Madison, Wisconsin, United States of America; University of Memphis, United States of America

## Abstract

Coordinated gene expression changes across the CNS are required to produce the mammalian maternal phenotype. Lateral septum (LS) is a brain region critically involved with aspects of maternal care, and we recently examined gene expression of whole septum (LS and medial septum) in selectively bred maternal mice. Here, we expand on the prior study by 1) conducting microarray analysis solely on LS in virgin and postpartum mice, 2) using outbred mice, and 3) evaluating the role of sensory input on gene expression changes. Large scale changes in genes related to neuronal signaling were identified, including four GABA_A_ receptor subunits. Subunits α4 and δ were downregulated in maternal LS, likely reflecting a reduction in the extrasynaptic, neurosteroid-sensitive α4/δ containing receptor subtype. Conversely, subunits ε and θ were increased in maternal LS. Fifteen K+ channel related genes showed altered expression, as did dopamine receptors Drd1a and Drd2 (both downregulated), hypocretin receptor 1 (Hcrtr1), kappa opioid receptor 1 (Oprk1), and transient receptor potential channel 4 (Trpc4). Expression of a large number of genes linked to developmental processes or cell differentiation were also altered in postpartum LS, including chemokine (C-X-C) motif ligand 12 (Cxcl12), fatty acid binding protein 7 (Fabp7), plasma membrane proteolipid (Pllp), and suppressor of cytokine signaling 2 (Socs2). Additional genes that are linked to anxiety, such as glutathione reductase (Gsr), exhibited altered expression. Pathway analysis also identified changes in genes related to cyclic nucleotide metabolism, chromatin structure, and the Ras gene family. The sensory presence of pups was found to contribute to the altered expression of a subset of genes across all categories. This study suggests that both large changes in neuronal signaling and the possible terminal differentiation of neuronal and/or glial cells play important roles in producing the maternal state.

## Introduction

The establishment of the maternal phenotype requires a coordinated suite of changes in numerous biological pathways, from endocrine signaling and metabolic activity to nervous system properties and adaptive behaviors [1]–[3]. Maternal behavior in many mammals is critical for the survival of offspring. In mice, this includes behaviors such as nest building, nursing, and protection of offspring [4]. The generation of effective maternal behavior also involves modulation of pathways related to bond formation and sociability, as the mother-infant relationship is the primary social bond in all mammalian species [5]. Additional emotional pathways altered in the postpartum state include fear, stress, and anxiety. The transition from a virgin to lactating state provides a unique and powerful opportunity to examine the fundamental neurophysiology of a range of emotional traits because the observed changes are naturally occurring.

Lateral septum (LS) is a brain region that is centrally featured in a network of structures known to influence social and parental behavior and emotional states [6], [7]. It has connections to the medial preoptic area, hypothalamus, amygdala, ventral tegmental area, periaqueductal gray, and receives input from medial prefrontal cortex [7]–[9]. The goal of this study was to identify gene expression changes occurring naturally in the LS of lactating outbred mice that may be important markers of the maternal phenotype. LS has been linked to certain aspects of maternal care, including offspring protection. Pharmacological manipulations of GABA_A_ receptors in LS alter offspring protection [10] and it has recently been demonstrated that the production of GABA is increased in the LS of postpartum mice [11]. The heteropentameric, ionotropic GABA_A_ receptor is assembled from a pool of 16 known subunits, resulting in a diversity of receptor subtypes with unique properties, pharmacological profiles, and distributions throughout the brain. This diversity provides a high degree of flexibility in signal transduction and allosteric modulation [12]–[14], but the dynamic regulation of GABA_A_ receptor subunits in LS of maternal mice has yet to be studied. This study therefore has a particular focus on investigating expression changes in GABA_A_ receptors themselves as a possible mechanism of modulating GABA signaling in the maternal LS.

We recently performed a gene expression study in the whole septum of maternal mice selectively bred for high offspring protection [15]. The present study used a similar microarray approach and quantitative real-time PCR to expand on that line of work by 1) utilizing a more specific dissection exclusively of LS, 2) using outbred mice to yield more natural and broadly applicable results, and 3) evaluating the effects of sensory input from interaction with pups on gene expression. While one component of the maternal phenotype is established initially by the actions of hormones during pregnancy, sensory inputs from both changes within the mother and from interactions with offspring additionally shape the maternal brain and parental behavior. This study evaluated the degree to which expression changes in the maternal LS require the continued presence of pups.

The transition from a virgin to lactating state involves many genes across numerous biological pathways. To address this, we utilized system level approaches to analyze microarray data and detect enrichment in sets of genes with related functions. Using these methods, we were able to identify functional themes in the expression profile of the maternal LS.

## Results

**Figure 1:**
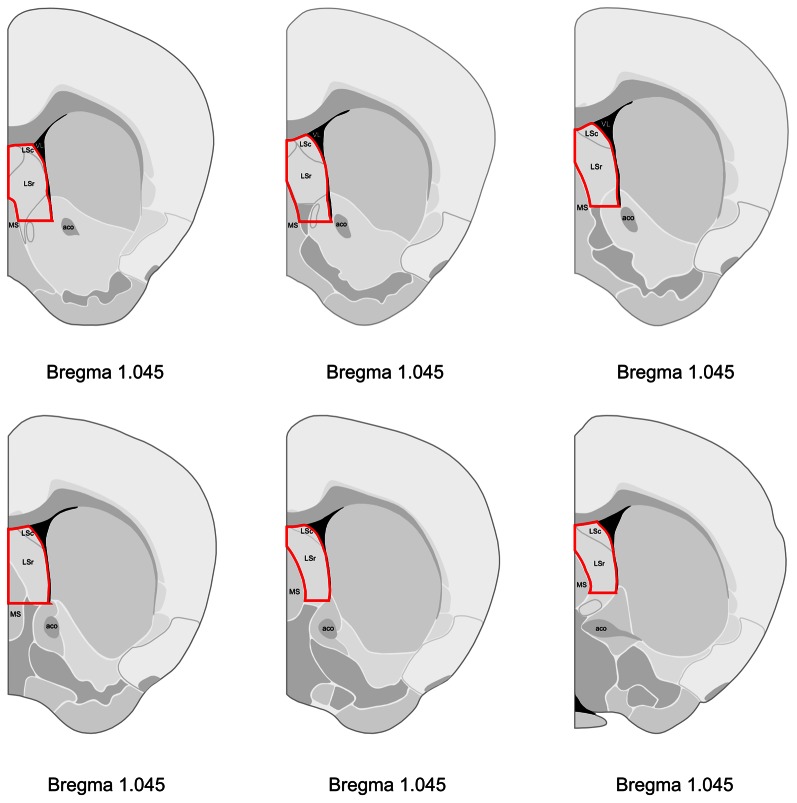
Schematic representation of the brain regions dissected for microarray and qPCR experiments. Dissections are outlined in red. Distance from Bregma in the rostrocaudal plane is indicated. Reprinted and modified from The Allen Mouse Brain Atlas (reference atlas version 1, 2008). Abbreviations: LSc, caudal part of the lateral septal nucleus; LSr, rostral part of the lateral septal nucleus; MS; medial septal nucleus; VL, lateral ventricle; aco, anterior commissure, olfactory limb.

Using Probe Logarithmic Intensity Error (PLIER) analysis, we identified 1,001 targets with significant expression differences (FDR-adjusted p<0.25) between the LS (Fig. 1) of lactating maternal and virgin mice. Of these, 809 corresponded to well-annotated genes. Table 1 presents the 100 most significant genes from this comparison, categorized by basic functional data from the PubMed database (http://www.ncbi.nlm.nih.gov/pubmed/) and GeneCards compendium (http://www.genecards.org). This table serves as a representative sample to highlight the microarray results, but many genes of biological interest and statistical significance cannot be included in such a short list. The full list of all 35,557 targets, their relative expression, and significance is presented in Table S1. Genes of interest reported in the microarray results include Gabra1 (FDR-adjusted p = 0.32, fold change 1.06), Gabra4 (FDR-adjusted p = 0.21, fold change 0.88), Gabrd (FDR-adjusted p = 0.11, fold change 0.75), Gabre (FDR-adjusted p = 0.12, fold change 1.38), Gabrq (FDR-adjusted p = 0.42, fold change 1.26), Cxcl12 (FDR-adjusted p = 0.25, fold change 1.14), Fabp7 (FDR-adjusted p = 0.20, fold change = 0.71), Gsr (FDR-adjusted p = 0.37, fold change 0.92), Pllp (FDR-adjusted p = 0.25, fold change 0.89), and Socs2 (FDR-adjusted p = 0.30, fold change 1.27).

**Table 1:** List of the 100 most significant gene expression differences between LS of lactating maternal mice and age-matched virgins.

Accession No.	Gene Symbol	Gene Title	Fold Change	
*Apoptosis, anti-apoptosis*				
NM_001079883	Bcl11b	B-cell leukemia/lymphoma 11B		0.79
NM_007537	Bcl2l2	BCL2-like 2		1.10
NM_009668	Bin1	bridging integrator 1		1.11
NM_011636	Plscr1	phospholipid scramblase 1		1.13
*Cell cycle, adhesion, division, death, differentiation and proliferation*				
NM_183187	Fam107a	family with sequence similarity 107, member A		1.17
NM_183183	Gprin3	GPRIN family member 3		0.79
NM_008258	Hn1	hematological and neurological expressed sequence 1		0.90
NM_001161535	Islr2	immunoglobulin superfamily containing leucine-rich repeat 2		0.85
NM_010660	Krt10	keratin 10		0.91
NM_178714	Lrfn5	leucine rich repeat and fibronectin type III domain containing 5		1.10
NM_177725	Lrrc8a	leucine rich repeat containing 8A		1.10
NM_028190	Luc7l	Luc7 homolog (S. cerevisiae)-like		1.08
NM_146061	Prr5	proline rich 5 (renal)		1.13
NM_011386	Skil	SKI-like		0.83
NM_011418	Smarcb1	SWI/SNF related, matrix associated, actin dependent regulator of chromatin, subfamily b, member 1		0.92
NM_133786	Smc4	structural maintenance of chromosomes 4		0.84
NM_001033272	Spata13	spermatogenesis associated 13		0.79
*Metabolic*				
NM_139306	Acer2	alkaline ceramidase 2		1.32
NM_177872	Adamts3	a disintegrin-like and metallopeptidase (reprolysin type) with thrombospondin type 1 motif, 3		0.74
NM_009625	Adcyap1	adenylate cyclase activating polypeptide 1		1.13
NM_018737	Ctps2	cytidine 5′-triphosphate synthase 2		1.10
NM_016699	Exosc10	exosome component 10		0.96
NM_021896	Gucy1a3	guanylate cyclase 1, soluble, alpha 3		0.81
NM_008509	Lpl	lipoprotein lipase		0.65
NM_172948	Mgat5b	mannoside acetylglucosaminyltransferase 5, isoenzyme B		0.91
NM_019840	Pde4b	phosphodiesterase 4B, cAMP specific		0.91
NM_172267	Phyhd1	phytanoyl-CoA dioxygenase domain containing 1		1.20
NM_029614	Prss23	protease, serine, 23		1.17
NM_027997	Serpina9	serine (or cysteine) peptidase inhibitor, clade A (alpha-1 antiproteinase, antitrypsin), member 9		0.52
*Phosphorylation, dephosphorylation*				
NM_177407	Camk2a	calcium/calmodulin-dependent protein kinase II alpha		0.95
NM_146125	Itpka	inositol 1,4,5-trisphosphate 3-kinase A		0.86
NM_175171	Mast4	microtubule associated serine/threonine kinase family member 4		1.13
NM_008587	Mertk	c-mer proto-oncogene tyrosine kinase		1.24
NM_016891	Ppp2r1a	protein phosphatase 2 (formerly 2A), regulatory subunit A (PR 65), alpha isoform		0.95
*Protein ubiquitination, deubiquitination*				
NM_172835	Peli3	pellino 3		1.09
NM_144873	Uhrf2	ubiquitin-like, containing PHD and RING finger domains 2		1.14
NM_001033173	Usp31	ubiquitin specific peptidase 31		1.14
NM_025830	Wwp2	WW domain containing E3 ubiquitin protein ligase 2		0.94
*Regulation of transcription*				
NM_010155	Erf	Ets2 repressor factor		0.88
NM_175660	Hist1h2ab	histone cluster 1, H2ab		0.68
NM_013550	Hist1h3a	histone cluster 1, H3a		0.78
NM_178203	Hist1h3b	histone cluster 1, H3b		0.77
NM_178204	Hist1h3d	histone cluster 1, H3d		0.78
NM_178204	Hist1h3e	histone cluster 1, H3d		0.78
NM_145073	Hist1h3g	histone cluster 1, H3g		0.77
NM_178206	Hist1h3h	histone cluster 1, H3h		0.77
NM_178207	Hist1h3i	histone cluster 1, H3i		0.78
NM_175662	Hist2h2ac	histone cluster 2, H2ac		0.88
NM_001013813	Maml2	mastermind like 2 (Drosophila)		0.87
NM_001033713	Mef2a	myocyte enhancer factor 2A		0.91
NM_001136072	Meis2	Meis homeobox 2		0.88
NM_172788	Sh3rf3	SH3 domain containing ring finger 3		1.11
NM_172913	Tox3	TOX high mobility group box family member 3		0.86
*Signal transduction*				
NM_133237	Apcdd1	adenomatosis polyposis coli down-regulated 1		0.88
NM_207655	Egfr	epidermal growth factor receptor		0.85
NM_008072	Gabrd	gamma-aminobutyric acid (GABA) A receptor, subunit delta		0.75
NM_175668	Gpr4	G protein-coupled receptor 4		1.19
NM_146072	Grik1	glutamate receptor, ionotropic, kainate 1		1.21
NM_181850	Grm3	glutamate receptor, metabotropic 3		0.87
NM_174998	Hpcal4	hippocalcin-like 4		0.89
NM_013568	Kcna6	potassium voltage-gated channel, shaker-related, subfamily, member 6		1.14
NM_010597	Kcnab1	potassium voltage-gated channel, shaker-related subfamily, beta member 1		0.86
NM_008427	Kcnj4	potassium inwardly-rectifying channel, subfamily J, member 4		0.72
NM_001081027	Kcnt2	potassium channel, subfamily T, member 2		1.15
NM_001081298	Lphn2	latrophilin 2		0.86
NM_024200	Mfn1	mitofusin 1		1.15
NM_152229	Nr2e1	nuclear receptor subfamily 2, group E, member 1		0.85
NM_178751	Orai2	ORAI calcium release-activated calcium modulator 2		0.86
NM_177411	Rab5b	RAB5B, member RAS oncogene family		0.91
NM_011243	Rarb	retinoic acid receptor, beta		0.66
NM_009107	Rxrg	retinoid X receptor gamma		0.71
NM_011313	S100a6	S100 calcium binding protein A6 (calcyclin)		0.81
NM_030889	Sorcs2	sortilin-related VPS10 domain containing receptor 2		0.91
NM_016908	Syt5	synaptotagmin V		0.94
NM_021344	Tesc	tescalcin		0.82
NM_011648	Tshr	thyroid stimulating hormone receptor		0.82
NM_031877	Wasf1	WASP family 1		0.91
*Translation*				
NM_178627	Poldip3	polymerase (DNA-directed), delta interacting protein 3		0.94
NM_024212	Rpl4	ribosomal protein L4		0.92
NM_007475	Rplp0	ribosomal protein, large, P0		0.90
NM_177214	Snrnp200	small nuclear ribonucleoprotein 200 (U5)		0.92
*Transport*				
NM_017391	Slc5a3	solute carrier family 5 (inositol transporters), member 3		1.16
NM_194355	Spire1	spire homolog 1 (Drosophila)		1.07
*Other*				
NM_133729	2610018G03Rik	RIKEN cDNA 2610018G03 gene		0.80
ENSMUST00000055842	Armcx4	armadillo repeat containing, X-linked 4		1.15
NM_027560	Arrdc2	arrestin domain containing 2		1.13
NM_028386	Asphd2	aspartate beta-hydroxylase domain containing 2		0.87
BC096543	B930095G15Rik	RIKEN cDNA B930095G15 gene		1.14
NM_027909	C2cd2l	C2 calcium-dependent domain containing 2-like		0.90
NM_030179	Clip4	CAP-GLY domain containing linker protein family, member 4		0.88
NM_146067	Cpped1	calcineurin-like phosphoesterase domain containing 1		0.88
NM_198866	Dbpht2	DNA binding protein with his-thr domain		0.85
NM_001134457	Fam55c	family with sequence similarity 55, member C		1.10
NM_178673	Fstl5	follistatin-like 5		1.16
NM_207222	Lmo3	LIM domain only 3		0.81
NM_001164805	Thsd7a	thrombospondin, type I, domain containing 7A		0.81
NM_198627	Vstm2l	V-set and transmembrane domain containing 2-like		0.90
NM_001081382	Zfp777	zinc finger protein 777		0.92
NM_013859	*Znhit2-ps*	zinc finger, HIT domain containing 2, pseudogene		0.90
NM_145456	Zswim6	zinc finger, SWIM domain containing 6		0.84

All expression changes in Table 1 have FDR-adjusted p-values less than 0.11. Expression is given as fold change in lactating maternal LS relative to virgin; numbers greater than 1.0 indicate increases in maternal mice.

**Figure 2:**
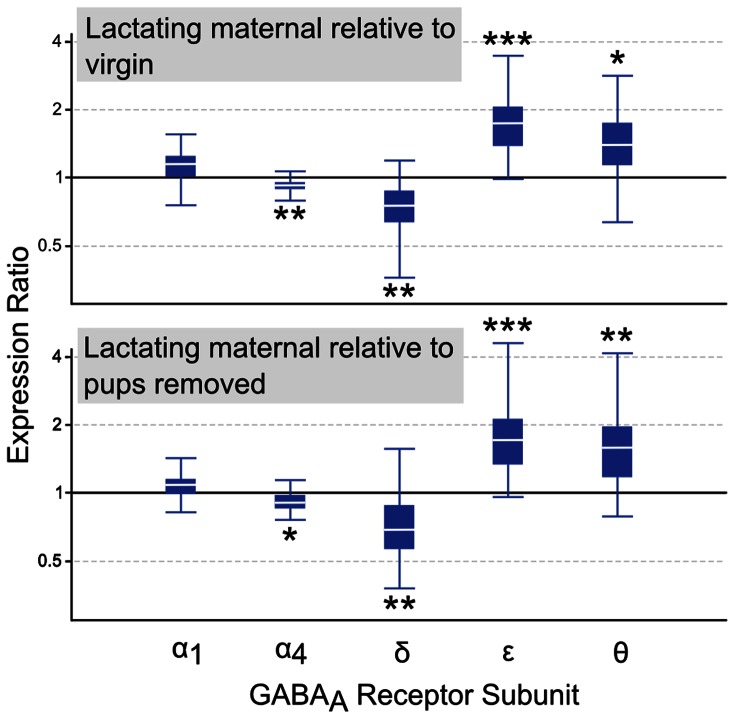
Quantitative real-time PCR analysis of GABA_A_ receptor subunit expression in lateral septum. Relative expression distribution (Y-axis) represented as a ratio of lactating maternal versus virgin (top panel, n = 8/group) and lactating maternal versus pups removed (bottom panel, n = 8/group), was normalized against two reference genes, Ppia and Ywhaz, and shown by box-and-whisker plots as medians (white lines), interquartile ranges (boxes), and ranges (whiskers). Ratios over one indicate genes that are more highly expressed in lactating maternal LS than in virgin or pups removed LS. *p<0.05; **p<0.01; ***p<0.001.

Numerous expression changes in GABA_A_ receptor subunits were confirmed by quantitative real-time PCR analysis (Fig. 2). For each subunit that exhibited significant expression changes, differences were found to be significant in lactating maternal LS relative to both virgin and pups removed groups. The α4 subunit was downregulated (p = 0.004 and p = 0.046), as was the δ subunit (p = 0.003 and p = 0.01). The ε subunit expression was elevated (p<0.001 and p = 0.001), along with the θ subunit (p = 0.023 and p = 0.001). The observed increase in α1 subunit expression did not reach significance (p = 0.077 and p = 0.092).

**Figure 3:**
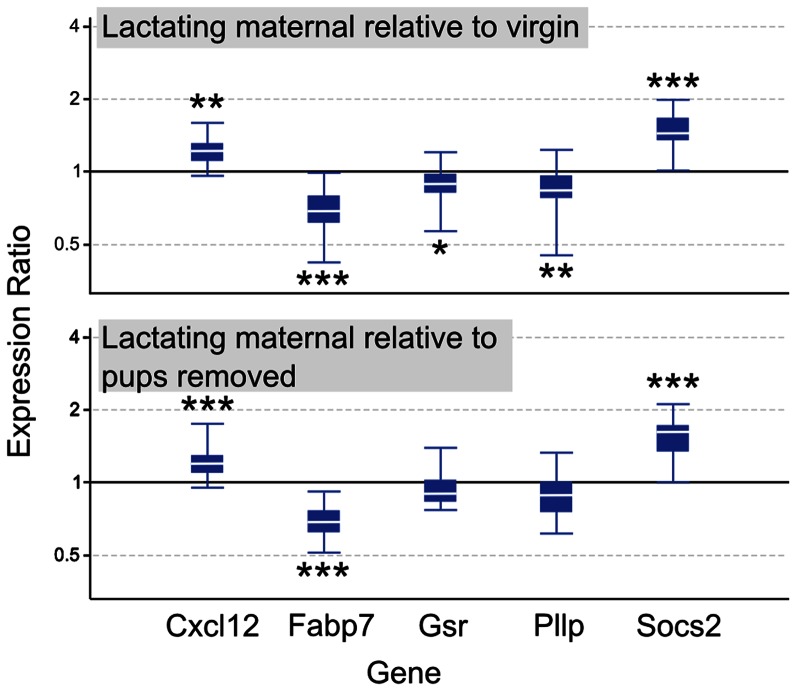
Quantitative real-time PCR analysis of gene expression in lateral septum. Relative expression distribution (Y-axis) represented as a ratio of lactating maternal versus virgin (top panel, n = 8/group) and lactating maternal versus pups removed (bottom panel, n = 8/group), was normalized against two reference genes, Ppia and Ywhaz, and shown by box-and-whisker plots as medians (white lines), interquartile ranges (boxes), and ranges (whiskers). Ratios over one indicate genes that are more highly expressed in lactating maternal LS than in virgin or pups removed LS. *p<0.05; **p<0.01; ***p<0.001.

Quantitative real-time PCR analysis was used to confirm expression changes observed in microarray analysis for several genes of interest (Fig. 3). These genes were selected not only for their statistical significance in the current microarray experiment, but because they have appeared with regularity in numerous sets of significant expression results conducted in our laboratory across various regions of the maternal mouse brain ([15], unpublished observations). In lactating maternal LS relative to virgin, Cxcl12 and Socs2 were upregulated (p = 0.002 and p<0.001), while Fabp7, Gsr, and Pllp were downregulated (p<0.001, p = 0.04, and p = 0.018). In lactating maternal LS relative to pups removed, expression of Cxcl12 and Socs2 were again significantly elevated (p = 0.001 and p<0.001). Fabp7 was downregulated (p<0.001), but decreases in Gsr and Pllp did not reach significance (p = 0.125 and p = 0.074).

**Figure 4:**
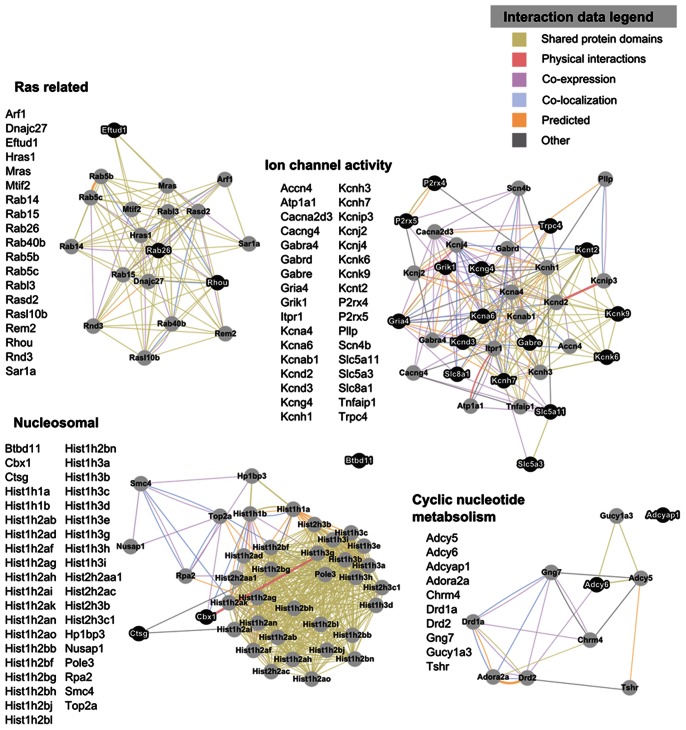
Gene clusters found to be enriched in the LS of lactating maternal mice relative to virgin mice as interaction networks. Gene lists for each cluster are presented to the left of their respective network visualization. Gene symbols in bold text are upregulated in lactating maternal LS relative to virgin, and are represented in the network images as dark nodes with white text. Non-bold gene symbols and grey nodes with dark text correspond to genes that are downregulated. The nature of the interaction data linking any two nodes is encoded by color. Distance between nodes is proportional to the strength of evidence for their interactions.

NIH DAVID’s functional annotation clustering tool identified enriched biological pathways among the 809 significant expression changes in lactating maternal LS relative to virgin (765 IDs recognized by DAVID). Figure 4 visualizes four gene clusters of interest with particularly high enrichment scores as networks of nodes connected by a variety of annotated interaction data. The ion channel activity network consists of 34 genes, of which 15 were upregulated and 19 were downregulated. The 19-gene network related to the Ras superfamily of small GTPases was primarily downregulated. The cyclic nucleotide metabolism network is comprised of 10 genes that influence the activity of adenylyl cyclase and the biogenesis of cyclic nucleotides. The nucleosome network is a large group of histone and histone-related proteins. It exhibits downregulation of the core histones H2a, H2b, H3, as well as two linker H1 histones. No H4 histones displayed significant expression changes. A more detailed expression data summary for enriched clusters is provided in Table S2.

**Figure 5:**
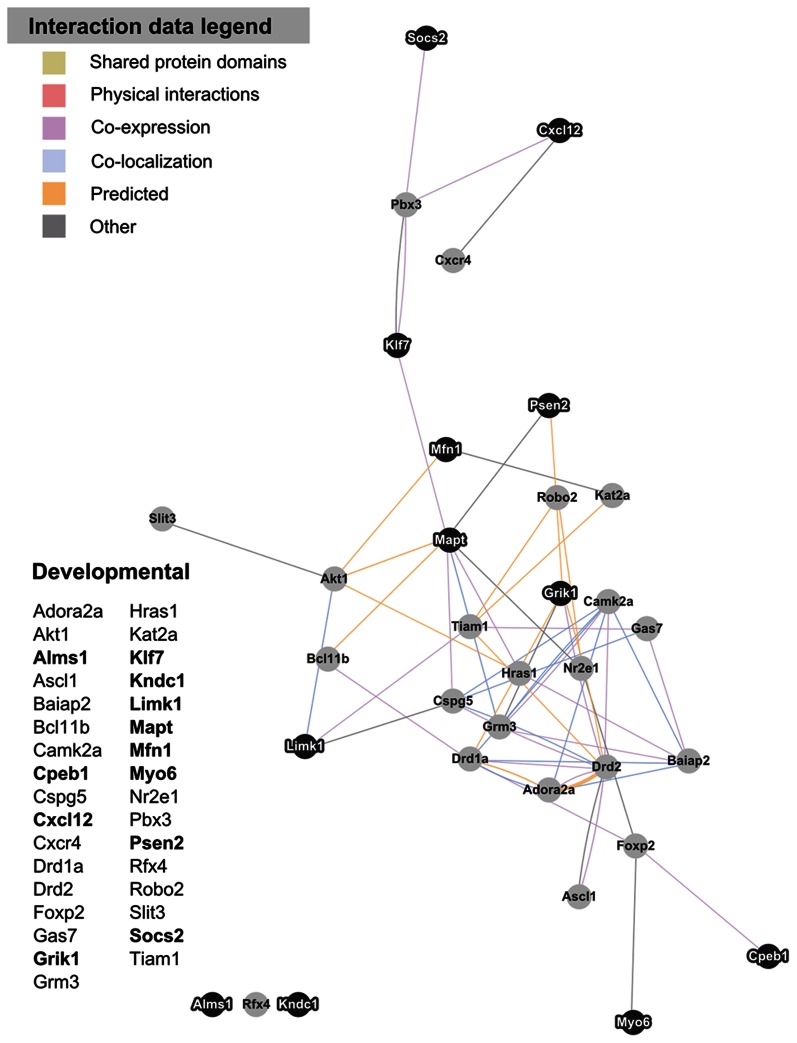
Cluster of genes enriched in maternal LS related to developmental processes and cell differentiation. Gene lists for the cluster is presented to the left of the network visualization. Gene symbols in bold text are upregulated in lactating maternal LS relative to virgin, and are represented in the network images as dark nodes with white text. Non-bold gene symbols and grey nodes with dark text correspond to genes that are downregulated. The nature of the interaction data linking any two nodes is encoded by color. Distance between nodes is proportional to the strength of evidence for their interactions.

In the 809 significant expression changes in maternal LS relative to virgin, NIH DAVID’s functional annotation clustering tool detected enrichment in numerous, small gene sets that each included genes linked to developmental processes and neuron/glial differentiation. We consolidated these developmentally related genes into a single, larger cluster that is visualized in Figure 5. Expression data for this cluster can be viewed in Table S2.

To assess which gene changes may be most strongly associated with continued sensory input from pups, we identified genes that exhibited significance in lactating maternal LS when compared to both virgin and pups removed groups. Table 2 presents the 69 genes for which expression changes relative to maternal were significant (FDR-adjusted p-values for both comparisons <0.25) and were in the same direction. This list therefore represents genes for which expression changes detected between lactating maternal and virgin LS were most closely mirrored in lactating maternal relative to pups removed. A full listing of all gene comparisons of maternal versus the pups removed group is also provided in Table S1.

**Table 2:** List of 68 genes that displayed significant expression changes in the LS of lactating maternal mice compared to both virgin and pups removed groups.

Accession No.	Gene Symbol		Gene Title	Fold Change (vs.pups removed)	Fold Change(vs. virgin)
*Cell cycle, adhesion, division, death, differentiation and proliferation*					
NM_170597	Creg2	cellular repressor of E1A-stimulated genes 2		0.90	0.92
NM_001166273	Cspg5	chondroitin sulfate proteoglycan 5		0.90	0.92
NM_183187	Fam107a	family with sequence similarity 107, member A		1.26	1.17
NM_011812	Fbln5	fibulin 5		1.24	1.19
NM_010218	Fjx1	four jointed box 1 (Drosophila)		0.84	0.87
NM_008538	Marcks	myristoylated alanine rich protein kinase C substrate		0.90	0.88
NR_033728	Nit1	nitrilase 1		1.06	1.08
NM_011386	Skil	SKI-like		0.87	0.83
*Metabolic*					
NM_001081204	B3galtl	beta 1,3-galactosyltransferase-like		0.91	0.92
NM_028979	Cyp2j9	cytochrome P450, family 2, subfamily j, polypeptide 9		1.18	1.14
NM_172948	Mgat5b	mannoside acetylglucosaminyltransferase 5, isoenzyme B		0.88	0.91
NM_172267	Phyhd1	phytanoyl-CoA dioxygenase domain containing 1		1.27	1.20
NM_029614	Prss23	protease, serine, 23		1.17	1.17
NM_001101430	Psmg4	proteasome (prosome, macropain) assembly chaperone 4		0.90	0.91
NM_133670	Sult1a1	sulfotransferase family 1A, phenol-preferring, member 1		1.34	1.30
*Phosphorylation, dephosphorylation*					
NM_009652	Akt1	thymoma viral proto-oncogene 1		0.93	0.92
NM_008587	Mertk	c-mer proto-oncogene tyrosine kinase		1.26	1.24
NM_016891	Ppp2r1a	protein phosphatase 2 (formerly 2A), regulatory subunit A (PR 65), alpha isoform		0.95	0.95
*Protein ubiquitination, deubiquitination*					
NM_173784	Ubtd2	ubiquitin domain containing 2		0.87	0.90
NM_144873	Uhrf2	ubiquitin-like, containing PHD and RING finger domains 2		1.13	1.14
*Regulation of transcription*					
BC024814	BC024814	cDNA sequence BC024814		0.87	0.90
NM_023876	Elp4	elongation protein 4 homolog (S. cerevisiae)		0.91	0.93
NM_133658	Ercc3	excision repair cross-complementing rodent repair deficiency, complementation group 3		0.93	0.93
NM_178213	Hist2h2ab	histone cluster 2, H2ab		0.89	0.87
NM_175662	Hist2h2ac	histone cluster 2, H2ac		0.90	0.88
NM_001033713	Mef2a	myocyte enhancer factor 2A		0.93	0.91
NR_030470	Mir669a-2	microRNA 669a-2		1.24	1.20
NM_181650	Prdm4	PR domain containing 4		0.93	0.95
NM_001166410	Rbm3	RNA binding motif protein 3		0.70	0.76
NM_001024918	Rfx4	regulatory factor X, 4 (influences HLA class II expression)		0.86	0.89
NM_029949	Snapc3	small nuclear RNA activating complex, polypeptide 3		1.06	1.07
NM_001168578	Tceal8	transcription elongation factor A (SII)-like 8		0.88	0.86
NM_172913	Tox3	TOX high mobility group box family member 3		0.87	0.86
NM_001164578	Tsr2	TSR2, 20S rRNA accumulation, homolog (S. cerevisiae)		0.94	0.91
NM_144546	Zfp119a	zinc finger protein 119a		0.87	0.89
*Signal transduction*					
NM_133237	Apcdd1	Adenomatosis polyposis coli down-regulated 1		0.83	0.88
NM_008533	Cd180	CD180 antigen		0.86	0.84
NM_009895	Cish	cytokine inducible SH2-containing protein		1.20	1.20
NM_010108	Efna3	ephrin A3		0.91	0.89
NM_207655	Egfr	epidermal growth factor receptor		0.89	0.85
NM_053072	Fgd6	FYVE, RhoGEF and PH domain containing 6		1.09	1.08
NM_175668	Gpr4	G protein-coupled receptor 4		1.18	1.19
NM_001081298	Lphn2	latrophilin 2		0.89	0.86
NM_001083897	Mpzl1	myelin protein zero-like 1		0.91	0.91
NM_019515	Nmu	neuromedin U		1.35	1.42
NM_152229	Nr2e1	nuclear receptor subfamily 2, group E, member 1		0.87	0.85
NM_178751	Orai2	ORAI calcium release-activated calcium modulator 2		0.86	0.86
NM_027571	P2ry12	purinergic receptor P2Y, G-protein coupled 12		0.88	0.87
NM_001042499	Rabl3	RAB, member of RAS oncogene family-like 3		0.92	0.91
NM_007706	Socs2	suppressor of cytokine signaling 2		1.32	1.30
NM_133789	Strn4	striatin, calmodulin binding protein 4		0.93	0.92
*Transport*					
NM_027560	Arrdc2	arrestin domain containing 2		1.11	1.13
NM_010634	Fabp5	fatty acid binding protein 5, epidermal		0.79	0.81
NM_021272	Fabp7	fatty acid binding protein 7, brain		0.69	0.71
NM_175112	Rae1	RAE1 RNA export 1 homolog (S. pombe)		0.94	0.92
NM_009155	Sepp1	selenoprotein P, plasma, 1		1.17	1.08
NM_178639	Sfxn5	sideroflexin 5		0.93	0.93
NM_146198	Slc5a11	solute carrier family 5 (sodium/glucose cotransporter), member 11		1.14	1.21
NM_138599	Tomm70a	translocase of outer mitochondrial membrane 70 homolog A (yeast)		0.90	0.88
*Other*					
NM_025675	Atpbd4	ATP binding domain 4		0.86	0.91
BC090976	BC022687	cDNA sequence BC022687		0.89	0.90
NM_001166164	Ccdc74a	coiled-coil domain containing 74A		0.89	0.89
NM_030179	Clip4	CAP-GLY domain containing linker protein family, member 4		0.91	0.88
NM_153507	Cpne2	copine II		0.91	0.92
NM_146067	Cpped1	calcineurin-like phosphoesterase domain containing 1		0.86	0.88
NM_026062	Fam69a	family with sequence similarity 69, member A		0.87	0.87
NM_001033550	Lrrc8b	leucine rich repeat containing 8 family, member B		0.92	0.92
NM_026845	Ppil1	peptidylprolyl isomerase (cyclophilin)-like 1		0.87	0.83
NM_024196	Tbc1d20	TBC1 domain family, member 20		0.90	0.89

The genes in Table 2 have FDR-adjusted p-values for each individual comparison under 0.25. Expression is given as fold change in lactating maternal LS relative to pups removed or virgin groups.

## Discussion

This study used Affymetrix microarray and quantitative real-time PCR to identify gene expression changes occurring naturally in LS of mice in association with the transition from a virgin to postpartum state at multiple levels of analysis, from single genes to enriched networks and biological pathways. Additionally, we evaluated the degree to which expression changes are dependent on the continued sensory input from pups. The results reveal numerous changes in genes of interest that may be of particular importance as markers of the maternal phenotype, including differential regulation of GABA_A_ receptor subtypes.

### Dynamic Regulation of GABA_A_ Receptor Subunits in Maternal LS

The GABA_A_ receptor was selected as a protein of interest based on previous studies suggesting the importance of GABA signaling in the LS for maternal behavior. Site-directed application of GABA_A_ receptor antagonist was shown to inhibit offspring protection in lactating maternal mice [10], and it has recently been demonstrated that glutamic acid decarboxylase (GAD) 65 and 67, the rate-limiting enzymes in the production of GABA, are upregulated in rostral LS of postpartum maternal mice [11]. The present study explores whether changes in GABA_A_ receptor expression might be a mechanism of regulating GABA signaling in LS.

In the microarray analysis, three GABA_A_ receptor subunits showed significant expression differences in lactating maternal LS relative to virgin, including δ, ε, and α4 (FDR-adjusted p<0.25). Several additional subunits, such as α1, ρ2, γ1, α5, θ, and β2, exhibited possible significance (PLIER p<0.05). The most ubiquitous and abundant GABA_A_ receptor subtype in the brain is composed of two α1, two β2, and one γ2 subunit [12], [14]. α1 and β2 displayed relatively small fold changes (1.06 and 1.05), while much more robust alterations were detected for δ (0.75), ε (1.39), α4 (0.88), and θ (1.26 (Table S1)). qPCR confirmation of the α1 result showed an increase that did not reach significance (p = 0.077). This suggests that the commonly found α1β2γ2 subtype is not dynamically regulated (at least at high levels) in the LS during the transition from a virgin to lactating maternal state. Conversely, qPCR confirmed significant expression changes for δ, ε, α4, and θ subunits (Fig. 2).

δ assembles with α4 endogenously to form a benzodiazepine insensitive receptor that mediates a tonic, rather than phasic, form of inhibitory current. α4/δ containing receptors have a high affinity for GABA, resist desensitization, and exhibit some degree of agonist-independent activity. They are located extrasynaptically and are positively modulated by the binding of neurosteroids [16]–[18]. Expression of the δ subunit has been shown to be dynamically downregulated in several brain regions in pregnant mice and is proposed to be a mechanism of maintaining a steady level of inhibition in the face of increasing progesterone associated with the prepartum period [19]. Our results provide evidence that a reduced sensitivity to neurosteroid influence on GABA signaling in the LS may also be important for maintaining the maternal phenotype after parturition. Neurosteroids can be synthesized in peripheral locations before crossing the blood brain barrier and are also produced locally in some regions of the brain by certain neurons and glia that exert autocrine and paracrine-like effects [20]. It is also possible that a reduction in tonic GABA inhibition could allow for the emergence of more precise rapid, synaptic transmission. One study has shown that transgenic mice expressing more extrasynaptic GABA_A_ receptors than their wild type counterparts exhibited greater tonic currents and smaller GABA-mediated mIPSCs [21]. If an inverse relationship between these two modes of signaling exists, then regulation of the relative amounts of synaptic versus extrasynaptic GABA_A_ receptors could have significant effects on the nature of net neuronal activity in the maternal LS.

### Additional Changes in Genes Related to Neuronal Signaling in Maternal LS

A large and diverse group of genes related to neuronal signaling was enriched in the maternal LS relative to virgin, including a large number of potassium channel related genes (Fig. 4). These channels are involved with many physiological functions, including regulation of neurotransmitter release and neuronal excitability. They also play a crucial role in shaping the action potential [22]–[24]. Regulation of potassium channel subunits appears to be complex; approximately half of potassium channel genes in this enriched cluster were upregulated in maternal LS compared to virgin, while the other half were downregulated. The ion channel cluster also includes genes involved in purinergic signaling, calcium channels, and glutamate signaling. The transient receptor potential cation channel, subfamily C, member 4 (Trpc4) was detected by microarray to be elevated by 13% in maternal LS compared to both virgin and pups removed groups (Fig. 4; Table S1). Trpc4 is interesting because it has a very restricted distribution in the brain and is most highly expressed in LS. Trpc4 and Trpc1 are the predominant Trpcs in LS, and may form heteromeric channels responsible for maintaining plateau potentials that can cause epileptic burst firing and even cell death in LS neurons [25]. Trpc4 knockout rats exhibit a phenotype of reduced social exploration and heightened social anxiety [26]. It has additionally been shown that Trpc4 is expressed in tyrosine hydroxylase positive dopamine neurons in the ventral tegmental area [27], demonstrating a possible link to reward circuitry. It is therefore possible that the dynamic regulation of Trpc4 expression in the postpartum LS could be a central mechanism of altering sociability in maternal mammals.

The modulatory neurotransmitter dopamine contributes to learning motivation and reward associated behaviors [28]. Dopamine receptors 1a (Drd1a) and 2 (Drd2) were downregulated in maternal LS relative to virgin (by 21% and 17%, respectively), but the functional significance of the changes is still be to be evaluated. There is growing evidence that Drd2 interacts with the adenosine A2a receptor (Adora2a) [29], which is also downregulated in the maternal LS. Hypocretin (orexin) receptor 1 (Hcrtr1) was 24% higher in maternal LS relative to virgin in the microarray results. Hypocretin is produced in lateral hypothalamic neurons that project to numerous brain regions, including the septum, and these neurons show altered activity in the postpartum state [30]. Hypocretin acting on Hcrtr1 influences arousal, vigilance, and feeding behavior [31]–[33] as well as maternal behaviors [34] .

The kappa opioid receptor (Oprk1) displayed a 17% decrease in maternal LS relative to virgin. Some studies report that site specific manipulations of Oprk1 can mediate anxiety-like behavior, the effects of social defeat stress, and the extent to which social play is rewarding [35]–[37]. Plasmolipin (Pllp, also known as plasma membrane proteolipid) is a proteolipid expressed in oligodendrocytes [38] and is a component of myelin, representing up to nearly 5% of the membrane [39]. Changes in Pllp expression, including the 17% reduction in maternal LS compared to virgin as detected by our qPCR analysis (Fig. 3) could influence myelination and action potential conductance. Pllp also makes up 1–2% of clathrin-coated vesicles which appear to target specifically to the synaptic plasma membrane [40]. Additionally, Pllp has been observed to increase dramatically in primary cultures during differentiation for embryonic rat neurons and neonatal rat glia [41]. Therefore, it appears to have roles in development, myelination, and synaptic function in the LS. Collectively, the striking enrichment in neuronal signaling and ion channel activity represents a significant alteration in basic signaling properties and neuronal excitability in the maternal LS.

### Large Expression Changes of Genes Related to Development and Cell Differentiation in Maternal LS

A number of studies have indicated plasticity of the maternal brain, and it is possible that the maternal state represents an endpoint in neuronal or glial differentiation. It could be that some cells do not fully differentiate until the postpartum state, and this contributes to the emergence of the maternal phenotype. We identified changes in a number of genes with strong ties to developmental processes and neuronal/glial differentiation in the maternal LS (Fig. 5). A subset of these genes were of interest (and confirmed by qPCR) because they had also been highlighted as showing altered expression in prior and ongoing maternal brain studies ([15], unpublished observations). Thus, these genes could provide key support for long lasting developmental changes in the maternal LS. A 21% increase in Chemokine (C-X-C motif) ligand 12 (Cxcl12, also known as SDF1-1) was detected by qPCR in the LS of lactating maternal mice relative to virgin (Fig. 3). Cxcl12 has been shown to be essential for the guidance of neuronal and glial stem cells in embryonic development [42], and has also been linked to angiogenesis in both embryo and tumor formation [43], [44]. Cxcl12 is unique among chemokines, which are commonly promiscuous in their receptor binding, in that it is highly specific to its receptor, Cxcr4. Interestingly, our microarray analysis reports that Cxcr4 is significantly downregulated in lactating maternal LS compared to virgin (Fold change 0.82, FDR-adjusted p = 0.13 (Fig. 5; Table S1)), opposite to the observed upregulation of the Cxcl12 ligand. While Cxcl12 and Cxcr4 are known to be stably expressed and function in mature cells, their apparent dynamic regulation in postpartum LS suggests that developmental processes with which they are involved could be of importance in shaping the maternal phenotype.

A highly significant 32% decrease in fatty acid binding protein 7 (Fabp7) was observed by qPCR in maternal LS relative to virgin (Fig. 3). Fabp7 is a brain-specific member of a family of long chain polyunsaturated fatty acid binding proteins. Polyunsaturated fatty acids, such as arachidonic acid and docosahexaenoic acid are important structural components of the developing brain [45]. Fabp7 is responsible for transporting these hydrophobic molecules through cytoplasmic environments to their ultimate membranous destination [46]. In addition to its role in development, it has been shown that a null mutation in Fabp7 results in a rodent phenotype characterized by elevated anxiety and fear memory [47]. The robust Fabp7 decrease in lactating maternal LS relative to virgin detected in the present study is in agreement with a previous experiment carried out with lactating maternal whole septum of a mouse strain previously selected for high offspring protection [15] and in hypothalamus of outbred mice [48]. This confirmation is notable because it demonstrates that the observed phenomenon is not likely strain specific and may occur in multiple brain regions. These results suggest that Fabp7 may play a role in developmental processes involved with plasticity in the maternal LS and actively mediate emotional changes associated with the postpartum period.

Cytokine signaling in the CNS influences how stem cells respond to hormones and plays an important role in the differentiation of neural progenitor cells into either glia or neurons [49]. A family of genes called “suppressors of cytokine signaling” (Socs) is known to be a negative regulator of such pathways [50]. Socs2 is the most abundant of these proteins in the CNS [51], and it is thought to mediate a negative feedback loop on messaging pathways downstream of growth hormone binding in the central nervous system. Stem cells cultured from Socs2 knockout mice produced 50% fewer neurons when induced to differentiate, and generated more astrocytic glial cells [52]. Conversely, Socs2 overexpressing stem cells yielded a higher than normal neuron to astrocyte ratio after differentiation. Socs2 was dramatically upregulated in maternal LS compared to both virgin and pups removed (46% and 53%; Fig. 3). The strength of developmental relevance in these microarray data provides some support for the idea that maternity can be viewed as another stage in the mammalian life cycle characterized by terminal differentiation of the CNS. If indeed these gene changes reflect developmental activity in the maternal LS, further anatomical and histological studies may be able to provide more direct evidence by visualizing structural changes involved in shaping the maternal brain.

### Additional Enrichment Findings in Maternal LS

Functional annotation clustering revealed a small cluster of genes influencing the synthesis of cyclic nucleotides (Fig. 4), with most members exhibiting downregulation in the maternal LS compared to virgin. Cyclic nucleotides play a central role in a variety of intracellular signaling pathways as second messengers [53], [54] and can modulate neuronal excitability via the binding of cyclic nucleotide gated (CNG) channels. CNG channels are well known for their role in sensory transduction in retinal and olfactory cells, but are also expressed widely in the mammalian CNS and are likely involved with synaptic plasticity and development [55], [56]. The enrichment of this gene cluster indicates that, in addition to altering ion channel activity directly through expression of the channels themselves, there may be an additional level of regulation facilitated by fluctuating levels of cyclic nucleotides available to cells in the maternal LS.

There was a large degree of enrichment in a nucleosomal gene cluster primarily composed of histone genes. These genes were almost exclusively downregulated, but it is not clear what implications this has on the function of the maternal LS. It is possible that a downregulation of histone mRNA may reflect changes in post-transcriptional processes that influence the stability of histone mRNA transcripts [57], [58]. The visualized cluster in Figure 4 shows a strong interaction between chromobox homolog 1 (Cbx1, also known as heterochromatin protein 1 β) and the H3 histone. Cbx1 plays a major role in regulating higher order chromatin structure and gene transcription [59]. Additionally, Cbx1^−/−^ knockout mice exhibit a lethal phenotype characterized by aberrant neocortical development with reduced proliferation of neuronal precursors, demonstrating developmental relevance [60]. While the significance of the nucleosomal gene cluster is not clear, the robustness and consistency of its enrichment is profound, and may influence chromatin remodeling in LS in the establishment of the maternal brain.

The Ras related gene cluster includes members of the Ras family, which is involved with many different cellular processes. It has been widely linked to tumor formation and has been shown to contribute in conjunction with thymoma viral proto-oncogene (Akt) signaling to glioblastoma formation in the brain [61]. All three members of the Akt family exhibited indications of altered expression in maternal LS compared to virgin in our microarray results (Table S1). In addition to cancer, certain Ras members of this enriched gene cluster also influence exocytosis and vesicle trafficking [62], [63].

### Expression Changes of Anxiety Related Genes in Maternal LS

A number of genes, such as the GABA_A_ receptors and other neuronal signaling genes have been linked to anxiety. Glutathione reductase (Gsr) is also linked to anxiety, and qPCR confirmed a 12% decrease in Gsr in the maternal LS compared to virgin (Fig. 3). Lentiviral *in vivo* overexpression of Gsr in the cingulate cortex of C57BL/6J mice results in significant increases in anxiety-related behavior [64]. The same study also showed that, across several strains of mice, Gsr activity was highest in the most anxious strains and lowest in the least anxious strains, suggesting relevance of Gsr activity to normal variation in anxiety. The transition from virgin to lactating maternal states involves a natural change in anxiety, in which postpartum mice respond less to general stressors [65]. The Gsr mRNA reduction we measured in maternal mice is in agreement with these observations, but whether these changes in LS are causally linked to altered anxiety is not known.

### Sensory Input Contributions to Gene Expression in Maternal LS

The present study revealed that a subset of genes was strongly influenced by the presence of pups. The maternal state can involve both short term and long term changes. Studies indicate that maternal characteristics are more strongly and stably expressed with increasing numbers of pregnancies and that this occurs with long lasting gene expression changes [66]–[68]. Among the significant 809 gene expression changes between maternal and virgin LS, 69 were found to also be significantly different (FDR-adjusted p<0.25) between maternal and pups removed LS (Table 2). For these genes, the removal of pups most fully restored virgin-like levels of mRNA even after the experience of mating, pregnancy, and parturition. We interpret these genes as being more dependent on the continued presence of pups, in contrast to genes that were differentially expressed between maternal and virgin LS but not between maternal and pups removed LS. This list represents an interesting subset of genes that are more environmentally malleable to the social aspects of maternity. While formal pathway analysis cannot be reliably conducted on such a small set of genes, it can be seen from the basic categorization in Table 2 that they span numerous functional groupings. Fabp7 and Socs2 are notable members of this subset, which suggests that the developmental processes with which they are involved may be ongoing and driven in the postpartum period by social interaction.

## Materials and Methods

### Animals

Outbred hsd:ICR female mice (*Mus domesticus*) (Harlan, Madison WI) were used for all experiments. Nulliparous animals were split into three age-matched groups (∼70 days of age at time of dissection), designated as lactating maternal, pups removed, and virgin. For mating, females in the lactating maternal and pups removed groups were housed in polypropylene cages with a breeder male for 2 weeks. Virgin females were concurrently co-housed with one another to provide similar levels of exposure to social stimuli. After the separation of breeder males, all females (pregnant and virgin) were housed individually and provided precut nesting material until dissections. Under this schedule, all females experienced similar levels of co-housing and single housing to minimize potential effects of isolation-induced stress. Cages were changed once per week until pups were born (postpartum Day 0), after which cages were not changed again for all animals until dissection. On Day 0, pups were culled, if necessary, to standardize litter size to eleven. For females in the pups removed group, pups were removed from the cage on postpartum Day 2. The pups removed group was included in the experimental design to provide insight as to whether or not continued sensory input from pups is required, in addition to parturition, to generate expression changes characteristic of the maternal phenotype. All animals were housed in the same room with cages of each experimental group positioned in an alternating fashion on the same shelves. A 12∶12 light/dark cycle with lights on at 06∶00 h CST was used. Female mice were provided with ad lib access to breeder chow (Harlan) and tap water. Procedures were performed in strict accordance with the guidelines of the National Institutes of Health Guide for the Care and Use of Laboratory Animals, and all studies were approved by the University of Wisconsin Animal Care and Use Committee.

### Tissue Collection and RNA Extraction

On postpartum Day 7, brains were removed from females in the lactating maternal and pups removed groups between 10∶00 and 12∶00 h. Brains from age-matched virgin females were collected on the same day, during the same time period. Dissections were alternated among groups so that an equal number of dissections from each group were performed. Animals were lightly anesthetized with isoflurane and decapitated. After decapitation, vaginal lavage was performed on virgin and pups removed females to determine their estrous state. All females in the pups removed group were diestrous, while virgin females exhibited variance in estrous states. To control for effects of estrous cycling on gene expression, only diestrous virgins were used for analysis [69], [70]. Tissue collection was performed as previously described [15]. Briefly; the whole brain was removed, snap frozen in isopentane on dry ice, and stored at −80°C until sliced. Brain sections were sliced in a cryostat (Leica, CM1850, Bannockburn, IL, USA) at 200 micron thickness and mounted on glass slides. Target tissue was extracted by micropunch technique [71]. Microdissection of frozen brain sections was performed with the Brain Punch Set (Stoelting, Wood Dale, IL, USA) under a dissecting microscope. LS, including caudal and rostral portions, was collected bilaterally from Bregma 1.10 to 0.14 mm (Fig. 1) and consolidated such that each animal yielded one sample of LS. Microdissections from eight animals in each group were flash frozen on dry ice and stored at −80°C until processing for gene array analysis or quantitative real-time PCR. Total RNA was extracted and prepared in matched triplets, one sample from each experimental group, using the Aurum Total RNA Fatty and Fibrous Tissue Kit (Bio-Rad, Hercules, CA, USA) in accordance with manufacturer’s instructions. After isolation, RNA integrity was assessed using Agilent RNA 6000 Nano Chips and the Agilent Bioanalyzer 2100 (Agilent Technologies, Palo Alto, CA). The purity and yield of RNA samples were determined with a NanoDrop 1000 spectrophotometer (Thermo Scientific, Wilmington, DE, USA). Purified total RNA was stored at −80°C until processing.

### High-Density Oligonucleotide Array Hybridization

Six out of eight samples collected for each group (n = 6 per group) were randomly selected for use in the microarray experiment. Microarray analysis was performed with the GeneChip Mouse Gene 1.0 ST array (Affymetrix, Santa Clara, CA, USA) using targets derived from total RNA isolated from LS as described above. cDNA for array hybridization was reverse transcribed from 200 ng of total RNA using an Ambion GeneChip WT Expression Kit (Ambion, Austin, TX, USA) in accordance with the manufacturer’s specifications. In short, total RNA was used to synthesize double-stranded cDNA, which was then used as a template to synthesize single-stranded cRNA. This cRNA was subsequently used as a template for one round of single-stranded cDNA synthesis, and the resulting DNA-RNA hybrids were then degraded with RNase H. Amplified single-stranded cDNA was fragmented and biotinylated with an Affymetrix WT Terminal Labeling Kit (Affymetrix, Santa Clara, CA, USA) according to the manufacturer’s instructions. Fragmented, labeled cDNA samples were hybridized with the arrays for 16 hours at 45°C. Hybridized arrays were washed, stained, and subsequently scanned at 570 nm on an Affymetrix GC3000 G7 Scanner. The Affymetrix Command Console v. 3.1.1.1229 was used to extract and process data from scans. cDNA synthesis, fragmentation, labeling, array hybridization, and scanning were performed by the Gene Expression Center at the University of Wisconsin-Madison.

### Probeset Level Summarization and Microarray Statistical Analysis

Probeset level summarization and data normalization were performed with the PLIER algorithm and GC bin background correction using Affymetrix Power Tools v. 1.12.0. The raw and summarized microarray data presented in this publication have been deposited in NCBI’s Gene Expression Omnibus [72], and are accessible through GEO Series accession number GSE43627. GEO reporting meets the requirements of the Minimum Information About a Microarray Experiment (MIAME). An array-specific, empirical Bayesian implementation of ANOVA was used in the BioConductor package limma v.3.6.9 [73] to perform inferential statistics on gene expression changes between groups (lactating maternal vs. virgin, and lactating maternal vs. pups removed). Nominal and false discovery rate (FDR) corrected p-values were calculated, and fold changes for each gene were calculated in Excel by dividing the limma-calculated average lactating maternal expression by the limma-calculated average expression of virgin or pups removed groups.

### Quantitative Real-time PCR (qPCR)

To confirm expression changes detected by microarray analysis, qPCR was performed on genes of interest (n = 8 per group). Target genes included five GABA_A_ receptor subunits; α1 (Gabra1), α4 (Gabra4), δ (Gabrd), ε (Gabre), and θ (Gabrq), as well as five genes currently being evaluated as potential markers of the maternal phenotype; chemokine (C-X-C) motif ligand 12 (Cxcl12), fatty acid binding protein 7 (Fabp7), glutathione reductase (Gsr), plasma membrane proteolipid (Pllp), and suppressor of cytokine signaling 2 (Socs2). Two stable reference genes were used to normalize relative expression results in genes of interest; Tyrosine 3-monooxygenase/tryptophan 5-monooxygenase activation protein, zeta polypeptide (Ywhaz), and peptidylprolyl isomerase A (Ppia). Primer information can be viewed in Table S3. A SuperScript III First-Strand Synthesis System for RT-PCR (Invitrogen, Carlsbad, CA, USA) was used to reverse transcribe 100 ng of RNA to cDNA in an Eppendorf MasterCycler Personal PCR machine (Eppendorf, Hamburg, Germany) with poly-T 20mer primers.

### Functional Annotation Clustering and Interaction Mapping

DAVID’s functional annotation clustering is a tool which analyzes enrichment in a variety of precompiled gene sets assembled by shared ontology, protein domains, and biological pathways within a given list of input genes. DAVID then groups related gene sets into clusters. Enrichment scores are generated by detecting overrepresentation of a cluster within the input list compared to a reference background [74]. Functional annotation clustering with high classification stringency was performed on the 809 genes with FDR-adjusted p-values less than 0.25 between the lactating maternal and virgin groups, as reported by the probeset level summarization. Of these 809 genes, DAVID recognized 765 of them in its analysis. For several significant clusters, the non-redundant list of gene members comprising them were used as input in GeneMANIA to visualize each cluster as a network of nodes connected by lines representing known interactions and similarities.

## Supporting Information

Table S1 **Full list of all microarray targets, their relative expression, and FDR-adjusted p-values.**
(XLSX)

Table S2 **Expression data summary for enriched gene clusters found in NIH DAVID functional annotation clustering.**
(XLSX)

Table S3 **Primers for genes of interest and reference genes used in real-time quantitative PCR experiments.**
(DOCX)
