# Supplementary material for: Large Scale Expression Changes of Genes Related to Neuronal Signaling and Developmental Processes Found in Lateral Septum of Postpartum Outbred Mice
Source: PLoS One. 2013 May 22;8(5):e63824. doi: 10.1371/journal.pone.0063824 (PMC3661729; doi:10.1371/journal.pone.0063824)
Supplement: Table S3 — Primers for genes of interest and reference genes used in real-time quantitative PCR experiments. (DOCX) [file pone.0063824.s003.docx]

| **Supplementary Table 3. Primers for genes of interest and reference genes used in real-time quantitative PCR experiments.** | | | | | |
| --- | --- | --- | --- | --- | --- |
| **Gene symbol** | **Full name** | **NCBI accession number** |  | **Primer (5’ -> 3’)** | **Primer position** |
| Gabra1 | Gamma-aminobutyric acid (GABA) A receptor, subunit alpha 1 | NM_010250.4 | Forward | TGCATTTAAGAACAGAACAGCC | 3271-3292 |
|  |  |  | Reverse | GGCAGCAGTGAAGTTATGAGAG | 3372-3351 |
| Gabra4 | Gamma-aminobutyric acid (GABA) A receptor, subunit alpha 4 | NM_010251.2 | Forward | GCTGCTGAAATGCTAATGTCC | 3381-3401 |
|  |  |  | Reverse | TCACTGACATTCCATCCAAGG | 3564-3544 |
| Gabrd | Gamma-aminobutyric acid (GABA) A receptor, subunit delta | NM_008072.2 | Forward | CTGCTACGTCTTTGTGTTTGC | 1055-1075 |
|  |  |  | Reverse | CCGTTTCTTCCTGTAGTCGG | 1130-1111 |
| Gabre | gamma-aminobutyric acid (GABA) A receptor, subunit epsilon | NM_017369.2 | Forward | GACCTGGTATGATGAGCGTC | 1625-1644 |
|  |  |  | Reverse | CATCCTTGTGGATGAGAGCC | 1794-1775 |
| Gabrq | gamma-aminobutyric acid (GABA) A receptor, subunit theta | NM_020488.1 | Forward | GGAGAGTTGTTGCCCGATAC | 1123-1142 |
|  |  |  | Reverse | TCCTGCACATTTGCTACCAC | 1177-1158 |
| Cxcl12 | Chemokine (C-X-C motif) ligand 12, isoform precursors gamma, beta, and alpha | NM_001012477.2  NM_013655.4  NM_021704.3 | Forward | CACGGCTGAAGAACAACAAC | 303-322 |
|  |  |  | Reverse | ACTTTAATTTCGGGTCAATGCAC | 353-331 |
| Fabp7 | Fatty acid binding protein 7, brain | NM_021272.3 | Forward | TAAGTCTGTGGTTCGGTTGG | 329–348 |
|  |  |  | Reverse | CCCAAAGGTAAGAGTCACGAC | 429–449 |
| Gsr | Glutathione reductase | NM_010344.4 | Forward | CCGCCTGAACACCATCTATC | 692-711 |
|  |  |  | Reverse | TTTCCCATTGACTTCCACCG | 803-784 |
| Pllp | Plasma membrane proteolipid | NM_026385.4 | Forward | GAGGCATAGAGGAAAGCGAA | 1589-1608 |
|  |  |  | Reverse | TGGACAAGACAAGGGCAAGA | 1799-1780 |
| Socs2 | Suppressor of cytokine signaling 2 | NM_001168655.1 | Forward | TGTGAGTCCCAACCTAGTGC | 1931-1950 |
|  |  |  | Reverse | TGTCCGTGGTCAGACAATTC | 2031-2012 |
| Ppia | Peptidylprolyl isomerase A | NM_008907.1 | Forward | TGCTGGACCAAACACAAACG | 347-366 |
|  |  |  | Reverse | GCCTTCTTTCACCTTCCCAAA | 446-426 |
| Ywhaz | Tyrosine 3-monooxygenase/tryptophan 5-  monooxygenase activation protein, zeta  polypeptide |  | Forward | TCCTTATTCCCTCTTGGCAG | 2432–2451 |
|  |  |  | Reverse | ATGGAAGCTACATTAGCGGTTT | 2502–2523 |
